# *Mycobacterium llatzerense*, a waterborne *Mycobacterium*, that resists phagocytosis by *Acanthamoeba castellanii*

**DOI:** 10.1038/srep46270

**Published:** 2017-04-10

**Authors:** Vincent Delafont, Ascel Samba-Louaka, Emmanuelle Cambau, Didier Bouchon, Laurent Moulin, Yann Héchard

**Affiliations:** 1 Laboratoire Ecologie et Biologie des Interactions, Equipes « Microbiologie de l’Eau » et « Ecologie, Evolution, Symbiose», Université de Poitiers, UMR CNRS 7267, F-86073 Poitiers, France; 2 Eau de Paris, Direction de la Recherche et du Développement pour la Qualité de l’Eau, R&D Biologie, 33, Avenue Jean Jaurès, F-94200 Ivry sur Seine, France; 3 APHP Groupe Hospitalier Saint-Louis Lariboisière-Fernand-Widal, Centre National de Référence des Mycobactéries et de la Résistance aux Antituberculeux; UMR1137 IAME, Inserm, Université Paris Diderot, F-75010 Paris, France

## Abstract

Nontuberculous mycobacteria (NTM) are environmental bacteria increasingly associated to public health problems. In water systems, free-living amoebae (FLA) feed on bacteria by phagocytosis, but several bacteria, including many NTM, are resistant to this predation. Thus, FLA can be seen as a training ground for pathogenic bacteria. *Mycobacterium llatzerense* was previously described as frequently associated with FLA in a drinking water network. The present study aimed to characterize the interactions between *M. llatzerense* and FLA. *M. llatzerense* was internalised by phagocytosis and featured lipid inclusions, suggesting a subversion of host resources. Moreover, *M. llatzerense* survived and even multiplied in presence of *A. castellanii*. Using a genomic-based comparative approach, twelve genes involved in phagocytosis interference, described in *M. tuberculosis*, were identified in the *M. llatzerense* genome sequenced in this study. Transcriptomic analyses showed that ten genes were significantly upregulated during the first hours of the infection, which could partly explain *M. llatzerense* resistance. Additionally, *M. llatzerense* was shown to actively inhibit phagosome acidification. In conclusion, *M. llatzerense* presents a high degree of resistance to phagocytosis, likely explaining its frequent occurrence within FLA in drinking water networks. It underscores that NTM should be carefully monitored in water networks to prevent human health concerns.

*Mycobacterium* is a genus that is composed of at least 170 species, among which are found highly pathogenic mycobacteria, such as *M. tuberculosis* and *M. leprae,* causing tuberculosis and leprosy, respectively. Besides these pathogens causing major health concerns for humans and cattle, are nontuberculous mycobacteria (NTM), a vast group encompassing all other species from this genus. NTM are ubiquitously found in environments such as soil and water^1^. In terms of public health, NTM occurrence in drinking water network is of particular concern, as it is now recognized that several species are human pathogens transmitted through water systems^2^. Despite efforts in drinking water purification processes, the prevalence of NTM in drinking water networks increased steadily during the last decade^3^. Concomitantly, the prevalence of NTM diseases among the general population has risen from 1.8 cases per 100 000 persons in 1980, to 4.1–7.2 per 100 000 in the 2000′s^4^,^5^,^6^. It has been proposed that the successful colonisation and persistence of NTM in water networks, is partially explained by beneficial interactions with eukaryotic hosts such as free-living amoebae^7^,^8^. Free-living amoebae (FLA) are unicellular eukaryotes ubiquitously found in drinking water networks, that feed mainly on bacteria by phagocytosis^9^,^10^. Due to their bacterivorous activity, FLA play an important role for bacterial population control^11^. While most of bacteria internalised by FLA are rapidly digested through a phagocytic process, some other bacteria acquired elaborate ways to survive this predation. These so-called amoebae-resisting bacteria (ARB) comprise many bacterial representative, the most extensively studied being the intracellular pathogen *Legionella pneumophila*^12^,^13^,^14^. The capacity of these ARB to survive FLA phagocytic process led to consider these eukaryotic microorganisms not only as predators, but also as a training ground for emerging pathogens, favouring the development of resistance mechanisms for intracellular persistence^15^. The tuberculous and leprous mycobacteria were previously shown to resist to FLA phagocytosis *in vitro*, as well as some NTM species, being thus considered as ARB^16^,^17^,^18^,^19^,^20^. Investigations on environmental NTM resistance to FLA underlined that most of the species tested were able to survive *in vitro* within *Acanthamoeba spp.*^19^.

Several *Mycobacterium* species were studied for deciphering cellular processes involved in phagocytosis resistance, although most of the existing literature is focused on the highly pathogenic *M. tuberculosis,* as recently reviewed^21^. Most notably, several genes coding proteins interfering with phagosome acidification and phagolysosomal fusion were identified in *M. tuberculosis*^22^,^23^,^24^,^25^. Focusing on NTM, many experiments involved *Mycobacterium marinum* as a surrogate for the pathogenic *M. tuberculosis* bacilli. In this context, it was shown that the type 7 secretion system variant ESX-1 was required for intracellular thriving, when infecting *Acanthamoeba*^26^,^27^. Resistance of environmental NTM to FLA was also investigated using *Mycobacterium avium,* the major slowly-growing species involved in NTM diseases^28^. During infection of both human macrophages and FLA, it was demonstrated that a species-specific pathogenicity island was involved in the successful invasion of host cells by *M. avium*^29^. Another NTM studied is *Mycobacterium abscessus*, the major rapidly growing mycobacteria involved in human diseases^30^,^31^. The recent sequencing of *M. abscessus* whole genome highlighted the presence of a horizontally acquired phospholipase C (PLC), that was involved in intracellular proliferation within *A. castellanii*^32^.

We recently reported the frequent occurrence of FLA-NTM association in the drinking water network of Paris, France, and identified *M. llatzerense* as a predominant NTM co-occurring with FLA^33^. This NTM species, phylogenetically related to the opportunistic pathogens of the *M. mucogenicum* group, has been repeatedly identified within drinking water networks^34^,^35^,^36^,^37^,^38^. The involvement of *M. llatzerense* was recently reported in immunocompromised patients, raising the possibility that it may be an opportunistic pathogen as well^39^,^40^,^41^.

In order to explain why and how this NTM co-exists with FLA in water networks, we investigated the interactions of *M. llatzerense* and FLA, and hypothesized that this species was able to resist amoebal phagocytosis. Thereby, our work aimed to understand how *M. llatzerense* is coping with the amoeba model *Acanthamoeba castellanii*. This FLA species was chosen in this study because it is among the most frequently encountered FLA in the drinking water systems, as well as in association with mycobacteria^9^,^33^. We thus highlighted the ability of *M. llatzerense* to persist and multiply in presence of *A. castellanii*. The subcellular localisation of *M. llatzerense* within the amoeba host was characterised. A comparative genomics approach, using newly sequenced *M. llatzerense* genome, was used to identify conserved genes based on their role in mycobacterial resistance to phagocytosis. The involvement of these genes was subsequently monitored, by quantifying transcription levels during the infection process. Finally, an investigation at the phenotypic level allowed us to quantitatively evaluate the presence and survival of *M. llatzerense* within intracellular acidic compartments.

## Results

### *M. llatzerense* is resistant to *A. castellanii* predation.

**Figure 1:**
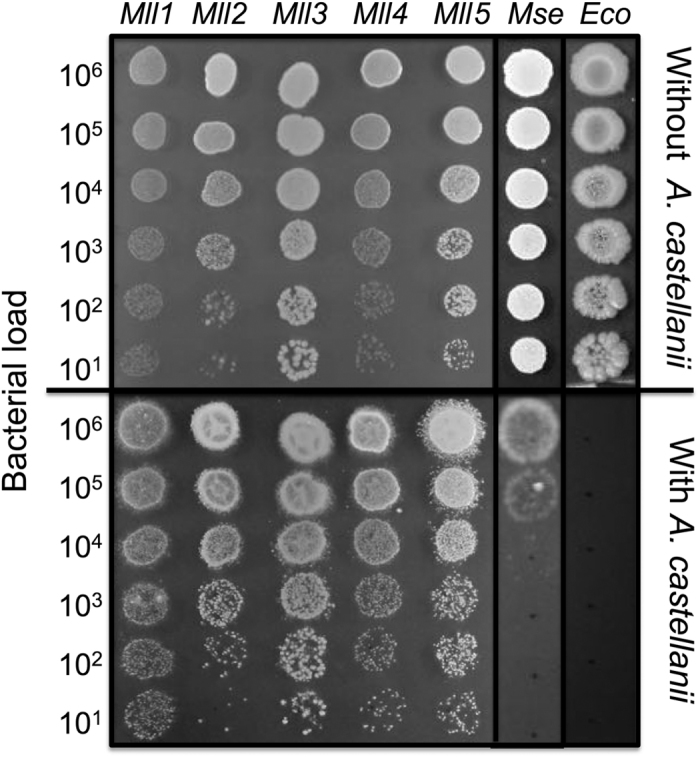
*M. llatzerense* resists *A. castellanii* predation. For the droplet test, bacterial suspension (10 μL) were spotted on Middlebrook 7H10, 10% OADC, with or without an *A. castellanii* lawn. Mll: *Mycobacterium llatzerense isolates EDP_1 to EDP_5; Ms: Mycobacterium septicum; Ec: Escherichia coli.*

In a previous study, we showed the frequent co-occurrence of *Mycobacterium llatzerense* (80% to 90% of identified NTM sequences) in association with FLA isolated from a drinking water network^33^. This finding encouraged a better characterisation of *M. llatzerense* interactions with *Acanthamoeba castellanii*. In this study, environmental isolates of *M. llatzerense* were collected from endpoint sites of the above-mentioned drinking water network, provided by treated groundwater. Their resistance to *A. castellanii* was determined through a droplet test, for comparing the ability of bacterial isolates to grow in presence or absence of *A. castellanii* seeded on the agar medium. This ability was assessed for five *M. llatzerense* environmental isolates, as well as a strain of *Mycobacterium septicum,* moderately resistant to *A. castellanii* predation, and isolated from the same drinking water network. *E. coli* was used as a control, as it is a commonly used food source for FLA (Fig. 1). The five environmental *M. llatzerense* isolates developed similarly with or without the presence of *A. castellanii* lawn on the nutritive medium (Fig. 1). In comparison, the ability of *M. septicum* to grow was strongly impaired in the presence of *A. castellanii*, while *E. coli* growth was completely inhibited (Fig. 1). Based on these observations, *M. llatzerense* displayed a resistance to *A. castellanii* predation. As no difference in *A. castellanii* resistance were observed between *M. llatzerense* environmental isolates, one representative isolate, *M. llatzerense* EDP_4, was selected for the subsequent experiments.

### *M. llatzerense* persists and grows in co-culture with *A. castellanii*

**Figure 2:**
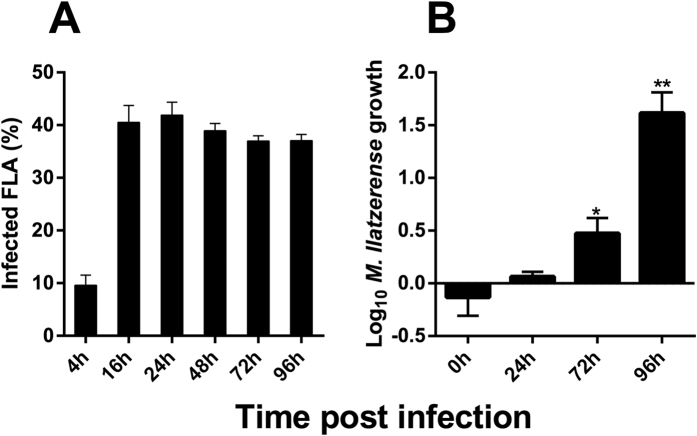
*M. llatzerense* persists and grows within *A. castellanii*. (**A**) Cell count of *A. castellanii* infected by *M. llatzerense* over-time. (**B**) CFU counts of *M. llatzerense* after *A. castellanii* infection at a MOI of 1 for 16 h. Each ratio was calculated with respect to the same condition without *A. castellanii*, where no significant growth was observed for *M. llatzerense* alone (data not shown). Statistical tests were performed using multiple unpaired t-tests (P < 0.05*P < 0.01**).

To confirm *M. llatzerense* resistance ability, its fate within *A. castellanii* was investigated during co-culture lasting up to 96 hours. This time course was chosen as it represents the longer incubation time inducing less than 10% *A. castellanii* encystment in the non-nutritive PAS buffer, the medium used for infection experiments. After 4 h of infection, about 10% of *A. castellanii* were infected, and reached 40% after 16 h (Fig. 2A). This infection rate was maintained without significant differences up to 96 h post infection. During the time course of the experiment, *M. llatzerense* viability was assessed using CFU counting. Viable mycobacteria were recovered at all time points of the experiments, demonstrating their resistance to *A. castellanii* phagocytosis (Fig. 2B). A significant growth was even observed, starting at 72 h (P < 0.05; unpaired t-test) and reaching a 1.5 log_10_ growth at 96 h post-infection (P < 0.01; unpaired t-test) (Fig. 2B).

### *M. llatzerense* is internalised by *A. castellanii*

**Figure 3:**
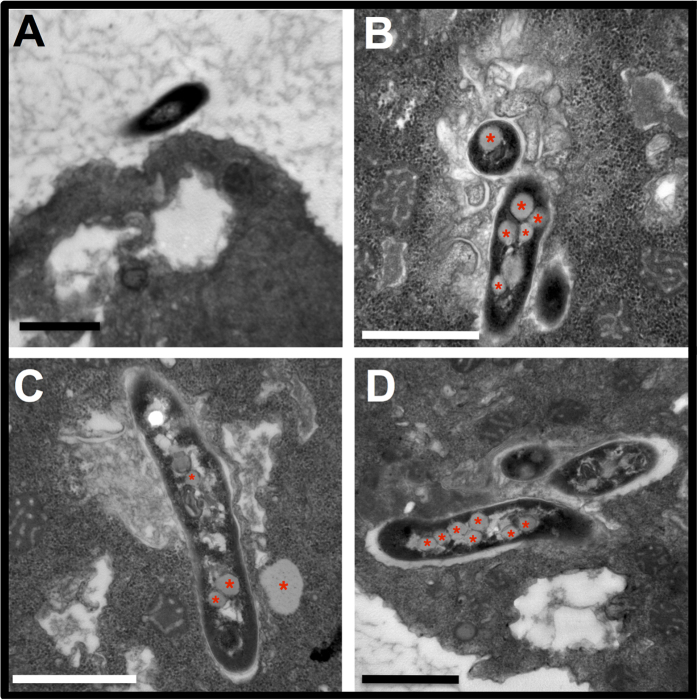
*M. llatzerense* EDP_4 is efficiently internalised by *A. castellanii.* Transmission electron microscopy of *A. castellanii* infected by *M. llatzerense* EDP_4 at a MOI of 1, at 4 h (**A,B**) 24 h (**C**) and 72 h (**D**) post infection. Bars represent 1 μm. Lipid bodies are indicated by asterisks on micrographs.

**Figure 4:**
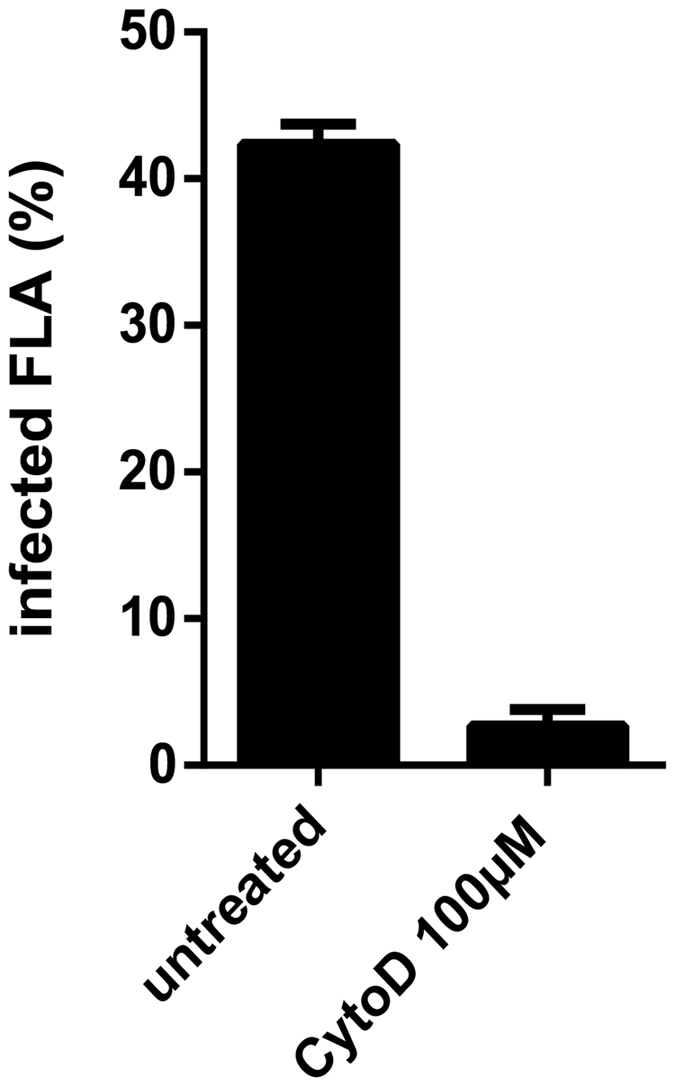
Cell count of *A. castellanii* infected with *M. llatzerense*_EDP_4, in presence (co-incubation) or in absence of cytochalasin D (CytoD), at a MOI of 10 for 4 h.

To gain insights into the interaction between *M. llatzerense* and *A. castellanii,* infected amoeba cultures were observed using transmission electron microscopy (TEM). *M. llatzerense* bacilli were mostly found intracellularly in vacuoles, which suggested an active internalisation process (Fig. 3). Starting from 4 h post infection, intracellular *M. llatzerense* harboured numerous inclusions into their cytoplasm, which were more frequently observed after 24 h and 72 h (Fig. 3B–D respectively). These inclusions were further confirmed to be of lipidic nature (supplementary Figure 1). In comparison, non-internalised bacilli did not harbour such inclusions (Fig. 3A). Lipid droplets from the host were also observed in the vicinity of *M. llatzerense*-containing vacuoles (Fig. 3C). Internalisation of bacteria by phagocytic cells such as FLA is mostly mediated by phagocytosis. Indeed, this was confirmed for *M. llatzerense*, as its internalisation was abrogated by the treatment of *A. castellanii* with cytochalasin D, a potent inhibitor of actin polymerisation (Fig. 4). Thus, it was shown that *M. llatzerense* internalisation by *A. castellanii* is indeed mediated by phagocytosis. This NTM species was able to persist intracellulary for at least 96 h.

### *M. llatzerense* shares phagosomal resistance genes with *M. tuberculosis*

In order to screen for conserved protein encoding genes involved in phagocytosis resistance, *M. llatzerense* whole genome was sequenced. The collection of 72′881′926 high quality bases allowed to draw the 6,7 Mb draft genome of *M. llatzerense,* assembled in 235 contigs. This genome is in the size range of previously described *Mycobacteria* genomes, comprised between 3.21 Mb and 7.3 Mb (*M. leprae* and *M. tusciae* respectively; Table 1). The genome size of *M. llatzerense* EDP_4 appeared slightly larger than another previously published genome from the same species (strain CLUC14; 6.1 Mb), while harbouring a similarly high GC content of 66.6%, versus 66.3% for *M. llatzerense* CLUC14. Further analyses between the two assemblies indicated an average nucleotide identity of 98.12%, hence confirming they belong to the same species (Supplementary figure 2)^42^. *M. llatzerense EDP_4* genome was screened for genes encoding proteins documented for their role in interfering with phagosome maturation in *M. tuberculosis,* using BLASTp searches. The *M. tuberculosis* H37rv genome was used for this comparative approach because it represents the most richly annotated genome to date within the *Mycobacterium* genus. In addition, gene functions were widely investigated using this strain, giving thus a highly valuable insight into the involvement of many genes in pathogenicity and intracellular survival^21^.

Among an initial set of thirteen coding genes, eleven highly similar, as well as one moderately similar genes were identified in *M. llatzerense* genome, and are described below (Table 2). Comparable results were obtained by using the *M. llatzerense* CLUC14 genome (Supplementary Table 2). Rv3707c, PPE10, Cut2, and GlyA1 were shown to be involved in in phagosome maturation blocking, as suggested by transposition mutagenesis in *M. bovis* Bacille Calmette-Guérin (BCG); mutant deleted for these genes were significantly enriched in acidified phagosome^43^. NdK of *M. tuberculosis* was shown to block phagosome maturation in murine macrophages through disruption of Rab5 and Rab7 GTPases, thus blocking phagosomes fusion with endosomal and lysosomal compartments^44^. Secreted phosphatases PtpA and PtpB were involved in mycobacterial pathogenesis; PtpA was notably characterised for inhibiting the recruitment of vacuolar-H +-ATPases machinery at phagosome membranes^25^. The SecA2 protein, part of the sec system, was also demonstrated essential for blocking phagosome maturation for *M. tuberculosis* infecting macrophages. PhoP and FbpA were both indirectly involved in blocking phagosomal maturation, as they regulate independently other virulence factors such as type 7 secretion systems (PhoP) or the cell wall lipid composition (FbpA)^23^,^45^. In addition, type 7 secretion system locus ESX-3 was identified in *M. llatzerense* genome, whose heterodimeric effector EsxG-EsxH was recently involved in disruption of *M. tuberculosis*-containing phagosomes addressing to the lysosome^24^.

### Phagosomal resistance genes are induced upon phagocytosis by *A. castellanii*

**Figure 5:**
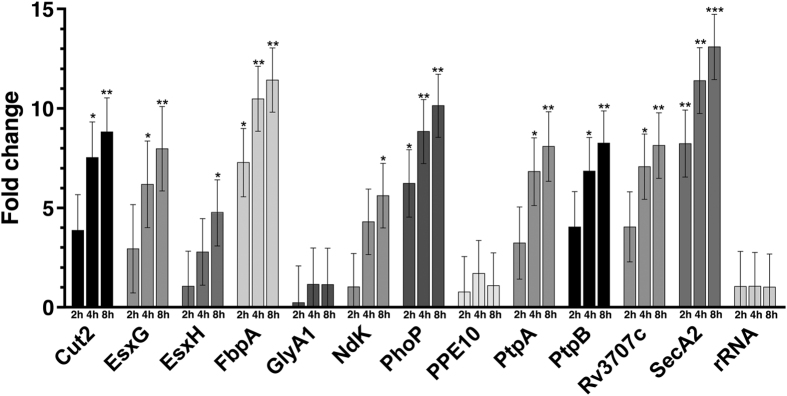
Fold change in transcription levels of selected conserved virulence factors at 2 h, 4 h and 8 h after *M. llatzerense* infection of *A. castellanii* at a MOI of 10, compared to 0 h condition. Statistical significance was assessed using multiple t-tests for comparison to control gene (16 S rRNA) at each time point. (P < 0.05*P < 0.01**P < 0.001***).

In order to investigate the potential role of these genes in *M. llatzerense* resistance to *A. castellanii*, their expressions were quantified by qRT-PCR at 0 h, 2 h, 4 h and 8 h post infection, corresponding to the early stage of *M. llatzerense* internalisation (Fig. 5). Overall, *M. llatzerense* internalisation significantly impacted the global transcriptional profile of selected genes (ANOVA, F = 85.608, df = 1–7, P = 0.001). After 2 h, only three of the selected genes were significantly upregulated, corresponding to *secA2* (8.23 folds), *fbpA* (7.28 folds) and *phoP* (6.23 folds). After 4 h, significant upregulations of transcription levels were observed for 8 other genes, namely *cut2, esxG, fbpA, phoP, ptpA, ptpB, rv3707c* and *secA2*. After 8 h, all genes except glyA1 and *ppe10* were significantly upregulated. Only genes that were upregulated after 2 h reached more than 10-times fold changes (fbpA, phoP and secA2; Fig. 5). In contrast, *glyA1* and *ppe10* did not show any significant change in transcription levels throughout the experiment. *The* early response of *M. llatzerense* to phagocytosis by *A. castellanii* is reflected by the stimulation of *fbpA, phoP* and *secA2* transcription. After 4 hours of infection, *M. llatzerense* transcriptional response to internalisation was prominent, while a late response is observed after 8 h through the significant upregulation of *esxH* and *ndK*. Taken together, the transcriptional increase of conserved virulence factors, shared between *M. tuberculosis* and *M. llatzerense*, is in favour of their involvement in the resistance to *A. castellanii* phagocytosis.

### *M. llatzerense* is not degraded by *A. castellanii* and resides in poorly acidified phagosomal compartments

**Figure 6:**
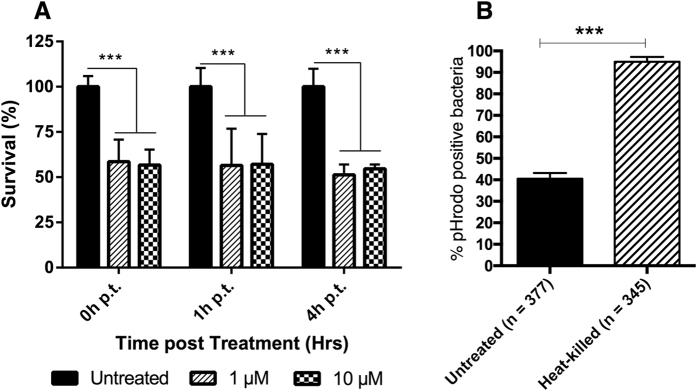
CFU count of *M. llatzerense* after infection of *A. castellanii* pre-treated or not with rapamycin. (**A**). Colocalisation of live or heat-killed *M. llatzerense* within intracellular acidic compartments labelled with pHrodo (**B**). Statistical tests were performed using unpaired t-tests (P < 0.001***).

In order to strengthen the results depicting significant changes of *M. llatzerense* gene expression during the early infection stage, we aimed to characterise its intracellular survival. Following internalisation, the resistance of *M. llatzerense* to *A. castellanii* degradation was assessed, by stimulating the autophagic machinery using rapamycin. Autophagy was described as a major pathway for blocking tuberculous mycobacteria proliferation in host cells, as its activation greatly affects mycobacteria survival^46^,^47^. *M. llatzerense* survival was monitored for up to four hours after rapamycin treatment. Bacterial viability significantly decreased at 0, 1 and 4 hours after rapamycin treatment, as half of the bacilli survived compared to control condition (Fig. 6A). While significant, even in condition where bacterial degradation through phagocytosis and autophagy were strongly stimulated, we did not reach *M. llatzerense* total elimination, confirming its strong ability to resist *A. castellanii* degradation.

In addition, the characterisation of *M. llatzerense*-containing phagosomes was undertaken, in order to correlate the pattern of gene expression with an observable phenotype. Therefore, the acidification of *M. llatzerense*-containing phagosomes was evaluated using a pHrodo dye, which act as a fluorescent indicator of acidic environment. When using alive *M. llatzerense* for infecting *A. castellanii*, 40.52 ± 2.65% of the bacterial cells were positive for pHrodo labelling (Fig. 6B, Supplementary figure 3). In contrast, infection of *A. castellanii* with heat-killed mycobacteria resulted in a significant increase of pHrodo labelled bacterial cells (94.95 ± 2.25%; P < 0.001, unpaired t-test). These results, coupled with previous observations at the transcription level, strongly suggest that *M. llatzerense* is actively blocking phagosomal maturation, ultimately resulting in *M. llatzerense* persistence in poorly acidified compartments.

## Discussion

NTM are frequently found in aqueous environments along with FLA, facing therefore a potentially strong predatory pressure, which suggests that NTM resistance stand as an evolutionary advantage to persist in such environments^7^,^10^,^48^,^49^. Accordingly, recent genomic findings indicated that mycobacteria may have maintained a sympatric life with protozoa such as FLA^50^. NTM, as environmental representatives of the *Mycobacterium* genus have been repeatedly reported to develop resistance to FLA predation^16^,^19^,^20^. As a fitting illustration of this statement, the recently characterised species *M. llatzerense* was recurrently found in drinking water, and in association with FLA. Therefore, we undertook the characterisation of resistance mechanisms employed by *M. llatzerense* to *A. castellanii* phagocytosis.

In the present study, the ability of *M. llatzerense* to persist and even grow in presence of FLA was described. As for other mycobacteria, *A. castellanii* internalised *M. llatzerense* through phagocytosis. Once internalised, *M. llatzerense* were found in vacuoles corresponding to phagosomes. The observed infectivity of *M. llatzerense* was comparable to those of *M. marinum* and *M. avium* infecting *Acanthamoeba spp.,* as reported previously^26^,^51^. While the resistance of a wide range of NTM has been observed, their capacity to grow in presence of amoebae has been much less described. Nonetheless, it was possible to evaluate that *M. llatzerense* growth in presence of *A. castellanii* was comparable to this of *M. avium* infecting the same FLA host, while higher than the growth observed for *M. fortuitum* and *M. marinum*^52^,^53^.

Interestingly, intracellular *M. llatzerense* harboured many intracytoplasmic lipid inclusions. Lipid bodies can represent a form of energy storage, but are also thought to be involved in host inflammatory and immune responses^54^. Similarly to the observations described in this study, it was shown that *M. marinum* was able to actively subvert host lipid bodies when infecting *D. discoideum*^55^. Although this type of observation needs to be repeatedly observed among various mycobacteria, it is likely that host lipid subversion represents a shared mechanism among the genus *Mycobacterium*.

In order to explore the genomic basis of *M. llatzerense* resistance to *A. castellanii*, a comparative genomic approach was undertaken. *M. llatzerense* whole genome sequencing revealed a large-sized 6.7 Mb genome, which is in accordance with its environmental and generalist lifestyle^56^. Screening for protein encoding genes involved in phagosome maturation blocking underlined the presence of twelve candidates shared with *M. tuberculosis* H37rv (reviewed in ref. 21). During *M. llatzerense* internalisation by *A. castellanii*, an early increase in *fbpA, phoP* and *secA2* transcription levels was observed. *FbpA* is supposedly involved in trehalose 6,6′ dimycolate (TDM) synthesis, an important surface glycolipid also designated as “cord factor”^45^. The significant upregulation of *fbpA* might lead to an enrichment of TDM, which was correlated with *M. tuberculosis* intracellular survival^57^. The early overexpression of *phoP* gene in *M. llatzerense* could suggest a role in blocking phagosome acidification. To support this hypothesis, *M. tuberculosis* mutants deficient for PhoP expression failed at blocking phagosome acidification, while still able to prevent phagolysosomal fusion^23^. The *secA2* gene showed one of the earliest and strongest overexpression following internalisation. This gene, as part of the sec system, was recently suggested to be involved in secretion of acid phosphatase PtpA and PtpB 22,25. In *M. llatzerense* the upregulation of *SecA2* transcription was parallel to those of *PtpA* and *PtpB*, which is in accordance with the proposed hypothesis. Indeed, a thorough investigation will be required to confirm the role of the Sec system in PtpA and PtpB secretion. Transcription analysis of *ndk* highlighted a late uperegulation. In *M. tuberculosis*, NdK was shown to inhibit phagosomal fusion with early endosomes and lysosomes^44^. The activity of T7SS ESX3 variant was indirectly monitored through *EsxG* and *EsxH* transcription, which were significantly upregulated at 4 h and 8 h post infection, respectively. These genes, encoding an ESX3 heterodimeric effector, were linked to the inhibition of phagolysosome fusion^24^. Structural and functional analyses of EsxG-EsxH heterodimer also suggested a role in iron and zinc scavenging^58^,^59^,^60^. *Cut2* and *Rv3707c* transcription was also significantly upregulated at 4 h post infection, however their roles are not yet clearly elucidated^43^.

In contrast to the significant changes observed in transcription levels of the other selected genes, both *glyA1* and *ppe10* were not differentially transcribed throughout the experiment. The roles for these genes have been identified by the characterisation of mutants unable to prevent phagosomal acidification^43^. PE-PPE proteins are thought to be involved in *Mycobacterium* pathogenicity^61^, therefore the expression profile of PP10 best BLAST hit identified in *M. llatzerense* seemed surprising. It is however worth noting that only a moderate sequence similarity (40% identity) was identified between PPE10 from *M. tuberculosis* and the corresponding protein in *M. llatzerense.* Furthermore, *M. llatzerense* EDP_4 was shown to harbour a total of 27 PE-PPE genes in its genome. It would be thus relevant to analyse the expression of these other genes during infection, in order to evaluate their importance in FLA phagocytosis resistance. So far the secreted hydroxymethyltransferase GlyA1 has only been described in *M. bovis* BCG for blocking phagosome maturation. As no significant difference in expression of *GlyA1* was observed in our study, we cannot confidently conclude on its role in the survival of *M. llatzerense* infecting *A. castellanii*. Transcription analyses indeed provide strong arguments in favour of the involvement of significantly modulated genes. However, it has to be kept in mind that functional analyses are required to confirm the importance of such genes in *M. llatzerense* resistance to *A. castellanii* phagocytosis, such as phenotypic characterisation of *M. llatzerense* survival within FLA when impaired for selected gene’s expression, through mutagenesis or interference experiments.

To complete transcription analyses, an investigation at the phenotypic level was performed. Most notably, a particular focus was given on the potential role of the autophagic machinery in modulating the infection. Autophagy, firstly described as a process maintaining cellular homeostasis through nutrients and organelles recycling, is also characterised as an antimicrobial mechanism, allowing the degradation of intracellular pathogens^62^. The autophagic machinery has been shown to play a role in the degradation of intracellular tuberculous mycobacteria, as it promotes phagosome maturation^46^. Unsurprisingly, autophagy induction in *M. llatzerense* – infected *A. castellanii* resulted in a significant decrease of bacterial survival. However, efficient *M. llatzerense* killing appeared nuanced, as 50% of intracellular mycobacteria were still viable after autophagy induction. The partial survival of *M. llatzerense* following autophagy activation might reflect its inherent resistance to advert conditions such as acidic and oxidative stresses. This finding is in accordance with a previous study describing *M. avium* ability to multiply within phagolysosomes, while *M. tuberculosis* was only able to persist in such condition^63^. In addition, by using a pH-sensitive fluorescent dye, it was possible to put in evidence that live mycobacteria were significantly less found in acidified compartments than heat-killed mycobacteria. This result strengthened the body of evidence regarding the ability of *M. llatzerense* to actively block *A. castellanii* phagocytosis process, and corroborate the transcription profiles of the analysed genes.

In conclusion, the work presented here provides insights into mechanisms employed by *M. llatzerense*, an environmental NTM, to resist *A. castellanii* predation. *M. llatzerense* was shown to infect efficiently *A. castellanii* through phagocytosis, remained viable and was able to grow for up to 96 h. *M. llatzerense* intracellular localisation within *A. castellanii* indicated persistence in vacuoles, as well as a potential subversion of host lipid metabolism. A comparative genomic approach identified twelve conserved virulence factors, shared between *M. llatzerense* and *M. tuberculosis*. Analyses of transcriptional profiles highlighted that all selected genes except *glyA1* and *ppe10* were significantly upregulated during the early stage of *A. castellanii* infection. This led to suggest that *M. llatzerense* is very likely able to actively hamper phagosomal acidification, while resisting to autophagy-induced advert conditions. Taken together, this study clearly defined *M. llatzerense* as an amoeba resistant bacteria. *M. llatzerense* was shown to establish an elaborate and efficient strategy not only to survive but to take advantage as well of the presence of FLA for its growth. This capability might very well explain the frequent recovery of this NTM in several drinking water networks.

## Methods

### Isolation and culture of microorganisms

NTM were isolated from Paris drinking water network. Briefly, water samples (1 L) were filtered through a 0.45 μm cellulose membrane, sonicated for 10 minutes, and decontaminated using 3% lauryl sulfate and 1% NaOH, as described previously^64^. Bacterial pellets were spread onto Lowenstein Jensen medium and incubated at 30 °C. Colonies were subcultured on Middlebrook 7H10 agar plates. To ensure the purity of isolated strains, isolates were decontaminated once again according to the Löwenstein method as described previously, and spread onto Middlebrook 7H10 plates supplemented with 10% OADC (Oleic acid, Albumin, Dextrose, Catalase; Becton-Dickinson)^65^. Ziehl-Neelsen cold staining was performed in order to confirm the presence of acid-fast bacilli. Briefly, heat-fixed samples were stained for 3 h in cold carbol Fuchsin. Samples were rinsed and treated using 25% sulphuric acid treatment for 45 s followed by absolute ethanol for 5 min. A counterstaining was performed by incubating samples with 0.2% methylene blue for 2 min. All mycobacteria isolates were maintained on Middlebrook 7H10 plates at 30 °C. *Escherichia coli* strain K-12 strain was grown and maintained on LB-agar (10 g/L tryptone, 5 g/L yeast extract, 10 g/L NaCl, 15 g/L agar) plates at 37 °C. *Acanthamoeba castellanii* strain ATCC 30234 was cultured axenically in PYG medium (20 g/L proteose peptone, 1 g/L yeast Extract, 1 g/L sodium citrate, 0.1 M glucose, 0.4 mM CaCl_2_, 4 mM MgSO_4_, 2.5 mM Na_2_HPO_4_, 2.5 mM KH_2_PO_4_, 50 μM Fe(NH_4_)_2_(SO_4_)_2_, pH 6.5) at 30 °C.

### Co-cultures and phagocytosis assays

*A. castellanii* monolayers, grown in PYG medium for 3 days, were collected, washed twice using PAS buffer (Page’s Amoeba Saline; 1 g/L sodium citrate, 0.4 mM CaCl_2_, 4 mM MgSO_4_, 2.5 mM Na_2_HPO_4_, 2.5 mM KH_2_PO_4_), adjusted to a final concentration of 2.5 × 10^5^ cells/mL and 1 mL was distributed in each well of a 12-wells plate. Mycobacteria grown for 4 days (*i.e.* early stationary phase) were used to infect *A. castellanii* at a multiplicity of infection (MOI) of 1 or 10 at 30 °C.

Droplet tests were performed by seeding homogeneously 4.10^6^ *A. castellanii* trophozoïtes on Middlebrook 7H10 agar supplemented with 10% (v/v) OADC (120X120 mm Petri dishes). Mycobacteria suspensions were prepared in PAS buffer, supplemented with 0.005% triton X-100 to avoid clumping, and homogenised by three passages using a 27 gauges needle. The suspension was spotted (from 1 × 10^8^ to 1 × 10^3^ bacteria/mL) on agar medium in presence or absence of *A. castellanii* as prepared previously, and incubated at 30 °C or 37 °C for optimal bacterial growth.

For the assessment of mycobacteria viability, *A. castellanii* monolayers in 12-wells plates were infected at a MOI of 1 for 16 h. Supernatant was discarded, cells washed twice and resuspended in 1 mL of fresh PAS buffer at 0, 24, 72 and 96 hours. Trophozoites were lysed and mycobacteria enumeration was performed as described above.

For evaluating the number of infected cells, *A. castellanii* monolayers in 12-wells plates were infected at a MOI of 1. Trophozoites were washed three times using PAS buffer before processing. Cells were centrifuged at 800 g for 5 minutes, supernatant was discarded, and the pellet resuspended in 50 μL of PAS, spread on a glass slide, heat-fixed and processed for Ziehl-Neelsen staining. Percentages of infected amoebae were calculated on the basis of at least 200 amoebae cells par slides, in a minimum of five different fields. In order to determine internalisation mechanisms of *M. llatzerense, A. castellanii* monolayers in 12-wells plates were infected at a MOI 10 for 4 h, in co-incubation with cytochalasin D at 100 μM in PAS buffer. For induction of autophagy *A. castellanii* trophozoïtes were infected at a MOI of 10 for 4 h, and treated for 1 h using 1 μM or 10 μM rapamycin in PAS.

### Evaluation of phagosomal acidification

*M. llatzerense* suspension prepared as described previously were pre-labelled using pHrodo^TM^ Red succinimidyl ester (ThermoFischer Scientific) following manufacturer recommendations, but excluding the methanol washing step. Briefly, bacterial suspensions were incubated for 1 h in 0.1 M sodium bicarbonate buffer containing 20 μM pHrodo^TM^ Red succinimidyl ester, in the dark. Bacteria were then pelleted by centrifugation at 12000 g for 5 min, and washed twice in PAS buffer supplemented with 0.005% triton X-100. In one condition, mycobacteria were killed before the initial incubation by heating the suspension at 85 °C for 15 min, in order to assess the importance of bacterial viability in subsequent analyses. *A. castellanii* monolayers were infected by either alive of heat-killed labelled mycobacteria at a MOI of 10, in 6 wells plates. After 2 h of incubation, trophozoites were detached, fixed with 2% paraformaldehyde for 15 min in the dark. Cells were pelleted by centrifugation at 800 g for 10 min, and resuspended in a 30 μL of SlowFade Diamonds antifade mountant (ThermoFischer Scientific) with Hoechst at a concentration of 400 ng/mL. Samples were examined using a confocal laser scanning microscope (SP8, Leica). To estimate the fraction of *M. llatzerense* residing in acidified phagosomes, a ratio was calculated based on the total number of bacteria (as detected by Hoechst staining) divided by the number of bacteria being labelled as well by pHrodo, which fluoresces only in acidic environment.

### Nucleic acid extraction

Isolated *M. llatzerense* colony was suspended in phosphate buffer saline, subsequently bead-beaten in tubes containing 500 mg of small diameter glass beads (100 μm) and 4 glass beads of 2 mm diameter (Sigma) using Fastprep apparatus for 30 s, put on ice for 1 min, and bead-beaten again using the same parameters. The suspension was then processed for DNA extraction using NucleoSpin Tissue kit (Macherey-Nagel), following manufacturer recommendations for bacterial DNA extraction.

For RNA extraction cells were collected, suspended in 400 μL of resuspension solution (sterile ultrapure water with 10% Glucose, 12.5 mM Tris, 5 mM EDTA, pH 7.6) and transferred to a tube containing 400 mg of small diameter glass beads (100 μm) and 500 μL of acid phenol. Samples were bead-beaten as described above and centrifuged for 5 min at 14000 g, 4 °C. Supernatants were kept on ice and transferred into clean tubes. 1 mL of TRIzol^®^ reagent (Life Technology) was added to each sample, and incubated at room temperature for 5 min. 100 μL of chloroform/isoamyl alcohol (24:1 v/v) was added to each sample, shaken vigorously, incubated for 1 min and centrifuged for 5 min at 14000 g. Aqueous phase was transferred into a new tube, treated again with 200 μL of chloroform/isoamyl alcohol as described above. The collected aqueous phase was washed with 500 μL of isopropanol for 1 h on ice and centrifuged at 16000 g for 30 min. Supernatants were discarded and pellets washed with 1 mL of 70% ethanol, centrifuged 15 min at 16000 g. Pellets were air dried and suspended 50 μL of ultrapure water, 7.5 mM sodium acetate, and 150 μL of absolute ethanol. Samples were incubated at −20 °C overnight and centrifuged for 30 min at 16000 g. Pellets were rinsed as described above, let air dried, and suspended in 30 μL of ultrapure water. RNA samples were subsequently treated using TURBO^TM^ DNase (Life Technologies) according to manufacturer recommendations, purified once again using phenol/chloroform and quantified by spectrophotometry. RNA were aliquoted, stored at −80 °C, and one aliquot of each condition was used immediately for reverse transcription, using GoScript^TM^ Reverse transcription System (Promega). The resulting cDNA were used for downstream quantification by qPCR.

### DNA amplification and quantification

Amplifications of targeted *loci* from total DNA were done by PCR. Mixtures were prepared in a final volume of 25 μL, containing 5 μL of 5x GC buffer, 400 nM of each deoxynucleotide triphosphate, 1,5 mM MgCl2, 500 nM of each primer, 0.5 U of Phusion polymerase (Thermo Scientific), 2.5 μL of template DNA and PCR grade water in sufficient quantity for 25 μL. Primers U341F (5′-CCTACGGGAGGCAGCAG-3′) U926R (5′-CCGTCAATTCMTTTRAGT-3′) and Myco F (5′-GGCAAGGTCACCCCGAAGGG-3′) Myco R (5′-AGCGGCTGCTGGGTGATC-3′) were used for partial amplification of *16 S* and *rpoB* genes, respectively^66^. PCR were carried out in a Master Cycler Thermocycler (Eppendorf). PCR mixtures were subjected to an initial denaturation at 98 °C for 30 sec, 35 cycles of denaturation at 98 °C for 10 sec, hybridization at 56 °C for 10 sec and elongation at 72 °C for 20 sec. A final extension step was applied at 72 °C for 5 min. Amplicons were checked on 1.5% agarose gel and stained with Midori Green (Nippon Genetics). cDNA was quantified by quantitative PCR (qPCR) using primers pairs targeting specific loci (Supplementary Table 1). Mixtures for quantification were performed in a final volume of 20 μL, containing 10 μL of 2X SYBR^®^ Green PCR Master Mix (Life Technologies), 250 nM of each primer, 2 ng of cDNA, and molecular grade water in sufficient quantity for 20 μL. Experiments were carried out in a Viia7 real time thermocycler (Life Technologies), consisting in an initial denaturation step at 95 °C for 10 min, 45 cycles of denaturation at 95 °C for 15 sec, annealing for 15 sec at 63 °C to 65 °C, and elongation at 72 °C for 20 sec. Fusion curves were collected for each experiments. Quantification of transcripts were performed by calculating fold changes using 2^−ΔΔCt^ method, corrected by qPCR efficiency for each gene, and normalised to the control condition at 0 h, as well as the control gene expression of 16S rRNA^67^. Statistical significance was assessed using multiple t-tests coupled with the Holm-Sidak method, as implemented in Graphpad Prism 6.

### Whole genome sequencing and annotation

Once identification and purity of mycobacterial isolates were confirmed by both 16 S and *rpoB* sequencing, total DNA were processed for whole genome sequencing, using GS junior 454 pyrosequencer (Roche). Library preparation, emulsion PCR and sequencing were performed following manufacturer’s protocols. Raw sequences were then assembled using GS denovo Assembler software (Roche). The assembly was uploaded for annotation using RAST online annotation tool^68^,^69^. Sequences resulting from the assembly are available on the NCBI database under accession number ASM186516v1.

### Electron microscopy

The localisation of *M. llatzerense* inside *A. castellanii* was investigated using transmission electron microscopy following the protocol described in^70^. Briefly, amoebae were carefully collected at 4 h, 24 h and 72 h after infection by *M. llatzerense* at a MOI of 1, and fixed in 3% glutaraldehyde and osmium tetroxide. Cells were dehydrated in successive baths of 50%, 70%, 90% and 100% acetone. Cells were embedded in araldite resin, and ultrathin section of 60 nm were cut and stained with uranylacetat and lead salts according to Reynolds. Ultrathin sections were observed using JEOL 1010 transmission electron microscope operating at 75 kV.

## Additional Information

**How to cite this article:** Delafont, V. *et al. Mycobacterium llatzerense*, a waterborne mycobacterium, that resists phagocytosis by *Acanthamoeba castellanii. Sci. Rep.* **7**, 46270; doi: 10.1038/srep46270 (2017).

**Publisher's note:** Springer Nature remains neutral with regard to jurisdictional claims in published maps and institutional affiliations.

## Supplementary Material

Supplementary Material

## Figures and Tables

**Table 1:** General genomic features of *M. llatzerense* EDP_4 draft genome.

Feature	*M.llatzerense* EDP_4
**Size**	6′697′820
**Numbers of contigs**	235
**N 50**	79′454
**Estimated coverage**	10,3X
**GC content**	66,3%
**Number of Protein Encoding Genes**	6243
**Gene density**	1072
**Number of tRNA**	46

**Table 2:** Genes coding for proteins involved in phagosomal maturation arrest, shared between *M. tuberculosis* H37Rv and *M. llatzerense* EDP_4.

Gene identification in *M. tuberculosis* H37Rv	Product	Best BLAST hit in *M. llatzerense EDP_4*	E-value	Identity (%)
*ndk* (Rv2445c)	Nucleoside diphosphate kinase	**PEG 1152**	1e-78	82
*ptpA* (Rv2234)	Low molecular weight protein tyrosine phosphatase	**PEG 5571**	2e-83	70
*ppe10* (Rv0442c)	PPE family protein	**PEG 150**	4e-21	40
*PE_PGRS30* (Rv1651c)	PPE family protein	*	*	*
Rv3707c	Hypothetical protein	**PEG 1752**	1e-175	71
*cut2* (Rv2301)	Serine esterase, cutinase family	**PEG 3132**	4e-80	60
*glyA1* (Rv1093)	Serine hydroxymethyl-transferase	**PEG 3860**	0	68
*phoP* (Rv0757)	DNA-binding response regulator	**PEG 1675**	1e-156	89
*fbp*A (Rv3804c)	Antigen 85-A precursor (Antigen 85 complex A)	**PEG 1462**	1e-171	72
*secA2* (Rv1821)	Protein export cytoplasm protein SecA ATPase RNA helicase	**PEG 4455**	0	83
*ptpB* (Rv0153c)	Protein tyrosine phosphatase	**PEG 5695**	1e-91	54
*esxG* (Rv0287)	ESAT-6 like protein EsxG	**PEG 4567**	1e-39	77
*esxH* (Rv0288)	ESAT-6-like protein EsxH, 10 kDa antigen CFP7	**PEG 4566**	1e-45	70

Identities and expectation values (E-values) are based on protein sequences comparison. Legend: *:No homologous protein found in *M. llatzerense* genome.
