# Supplementary material for: Mycobacterium llatzerense, a waterborne Mycobacterium, that resists phagocytosis by Acanthamoeba castellanii
Source: Sci Rep. 2017 Apr 10;7:46270. doi: 10.1038/srep46270 (PMC5385496; doi:10.1038/srep46270)
Supplement: Supplementary Material [file srep46270-s1.pdf]

Mycobacterium llatzerense, a waterborne *mycobacterium*, that resists phagocytosis by *Acanthamoeba castellanii*

Vincent Delafont[1,2,•], Ascel Samba-Louaka[1], Emmanuelle Cambau[3], Didier Bouchon[1], Laurent Moulin[2], Yann Héchard[1,*]

1 Laboratoire Ecologie et Biologie des Interactions, Equipes « Microbiologie de l'Eau » et « Ecologie, Evolution, Symbioses », Université de Poitiers, UMR CNRS 7267, F-86073 Poitiers, France.
2 Eau de Paris, Direction de la Recherche et du Développement pour la Qualité de l'Eau, R&D Biologie, 33, Avenue Jean Jaurès, F-94200 Ivry sur Seine, France
3 APHP Groupe Hospitalier Saint-Louis Lariboisière-Fernand-Widal, Centre National de Référence des Mycobactéries et de la Résistance aux Antituberculeux, ; UMR1137 IAME, Inserm, Université Paris Diderot, F-75010 Paris, France
• Current address: Division of Microbial Ecology, Department of Microbiology and Ecosystem Science, University of Vienna, Vienna, Austria

Supplementary information

Supplementary table 1: Primers designed in this study for targeting *M. llatzerense*'s selected conserved virulence factors.

Primer	Sequences (5'-3')	Target
Mll_peg1152_ndk_F	AGCTGGCGACCAAGCACTAC	*M. llatzerense ndK gene*
Mll_peg1152_ndk_R	TCGACGATGGCAGCCACAAC	
Mll_peg5571_ptpa_F	TCCGCATGATGCGCTCGTTC	*M. llatzerense ptpA gene*
Mll_peg5571_ptpa_R	AATCGTCGATGCCGCCGTAG	
Mll_peg150_PPE10_F	TGGCAATGCCGGTGTGGATG	*M. llatzerense ppe10 gene*
Mll_peg150_PPE10_R	AGCAAGGCCGAATGCACCTC	
Mll_peg1752_Rv3707c_F	GTTCCTGGCACATCGCAACTTC	*M. llatzerense rv3707c gene*
Mll_peg1752_Rv3707c_R	AGTTCTCCCGGTTGATGCTCAC	
Mll_peg3132_cut2_F	GCTCGGCGGTTACTCATTGG	*M. llatzerense cut2 gene*
Mll_peg3132_cut2_R	CGGGCTGTTGAAGGTGAACG	
Mll_peg3860_glya1_F	GGCTCGGTGATGACCAACAAG	*M. llatzerense glyA1 gene*
Mll_peg3860_glya1_R	GATGGCGAGTTGCTCTGTGAC	
Mll_peg1675_phop_F	ACCCACGAGGTGTGGAAAG	*M. llatzerense phoP gene*
Mll_peg1675_phop_R	GCCCGCGTTGATGATGAAG	
Mll_peg1462_fbpa_F	GCCCTTCCTGGTCAACATCTC	*M. llatzerense fbpA gene*
Mll_peg1462_fbpa_R	ATGTCGTCGGCCTTGTAGC	
Mll_peg4455_seca2_F	CACGCGAAACTGGTCAAGG	*M. llatzerense secA2 gene*
Mll_peg4455_seca2_R	GTCGTTCTTGGCGTTCAGC	
Mll_peg5695_ptpb_F	TTTCGATGCTCGCCGACTCC	*M. llatzerense ptpB gene*
Mll_peg5695_ptpb_R	TGAATCCGGTGCGGTCCTTG	
Mll_peg4567_esxg_F	CTCGGCAGTCGCGTTCCAG	*M. llatzerense esxG gene*
Mll_peg4567_esxg_R	GTCGAGCAGGGCGTTGACC	
Mll_peg4567_esxg_F	TGGCAGGCCCAGTGGAAC	*M. llatzerense esxH gene*
Mll_peg4567_esxg_R	TCGTGGGTGGTCGCCATC	
Mll_16S_F	GGCCTTCGGGTTGTAAACCTC	*M. llatzerense 16S rRNA gene*
Mll_16S_R	GTAGTTGGCCGGTGCTTCTTC	

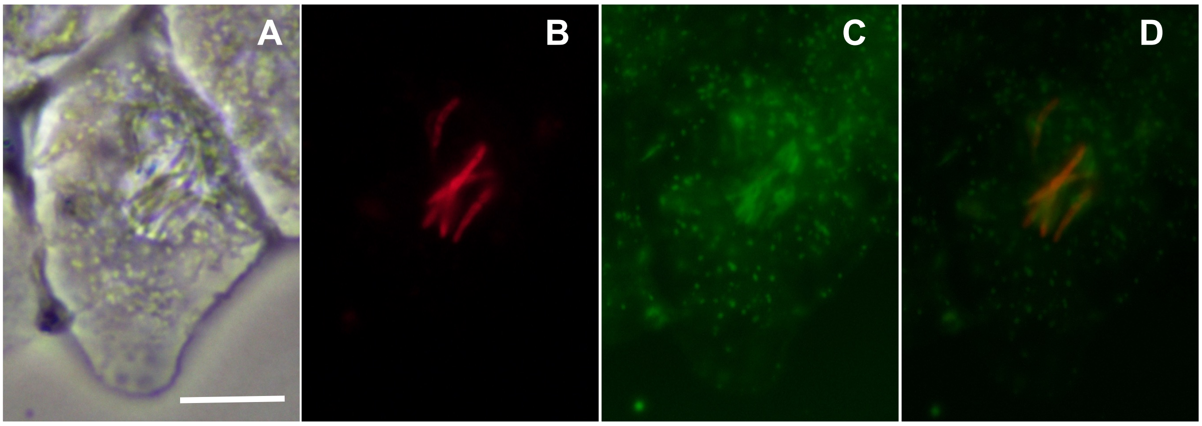

Supplementary figure 1: Co-localisation of amobeal lipid bodies with *M. llatzerense*. Phase contrast of an A. castellanii trophozoite infected by M. llatzerense (A). M. llatzerense expressing the fluorescent protein mcherry (B). Fluorescent labelling of lipid bodies of amoebal origin using Bodipy (C). Overlay of fluorescent channels, highlighting the colocalisation of lipid bodies with mycobacteria (D). *M. llatzerense* transformed as described previously with the plasmid pCHERRY3, and were used to infect A. castellanii at a MOI of 10^1. *A. castellanii* cultures were incubated prior to the infection with 10 µM Bodipy 493/503 in PAS buffer supplemented with palmytic acid, as described by Barisch and colleagues[2]. After 4 hours of infection, samples were fixed using 4% PFA, and observed using epifluorescence microscope. Scale bar: 2.5 µm.

Suuplementary table 2: Genes coding for proteins involved in phagosomal maturation arrest, shared between *M. tuberculosis* H37Rv and *M. llatzerense* CLUC14[3]. Identities and expectation values (E-values) are based on protein sequences comparison.

Gene identification in *M. tuberculosis* H37Rv	Product	Best BLAST hit in *M. llatzerense CLUC14*	E-value	Identity (%)
***Ndk* (Rv2445c)**	Nucleoside diphosphate kinase	**KIU14534**	2e-78	82
***PtpA* (Rv2234)**	Low molecular weight protein tyrosine phosphatase	**KIU15786**	3e-81	69
***PPE10* (Rv0442c)**	PPE family protein	**KIU17922**	8e-16	36
***PE_PGRS30* (rv1651c)**	PPE family protein	*	*	*
**Rv3707c**	Hypothetical protein	**KIU15472**	6e-175	72
***Cut2* (Rv2301)**	Serine esterase, cutinase family	**KIU18478**	3e-80	61
***GlyA1* (Rv1093)**	Serine hydroxymethyl-transferase	**KIU15277**	0	69
***PhoP* (Rv0757)**	DNA-binding response regulator	**KIU14827**	1e-155	90
***FbpA* (Rv3804c)**	Antigen 85-A precursor (Antigen 85 complex A)	**KIU14430**	5e-179	73
***SecA2* (Rv1821)**	Protein export cytoplasm protein SecA ATPase RNA helicase	**KIU16850**	0	83
***PtpB* (Rv0153c)**	Protein tyrosine phosphatase	**KIU14697**	2e-91	54
***EsxG* (Rv0287)**	ESAT-6 like protein EsxG	**KIU16620**	7e-41	78
***EsxH* (Rv0288)**	ESAT-6-like protein EsxH, 10 kDa antigen CFP7	**KIU16619**	3e-45	70

Legend: *: no homologous protein found in *M. llatzerense* genome

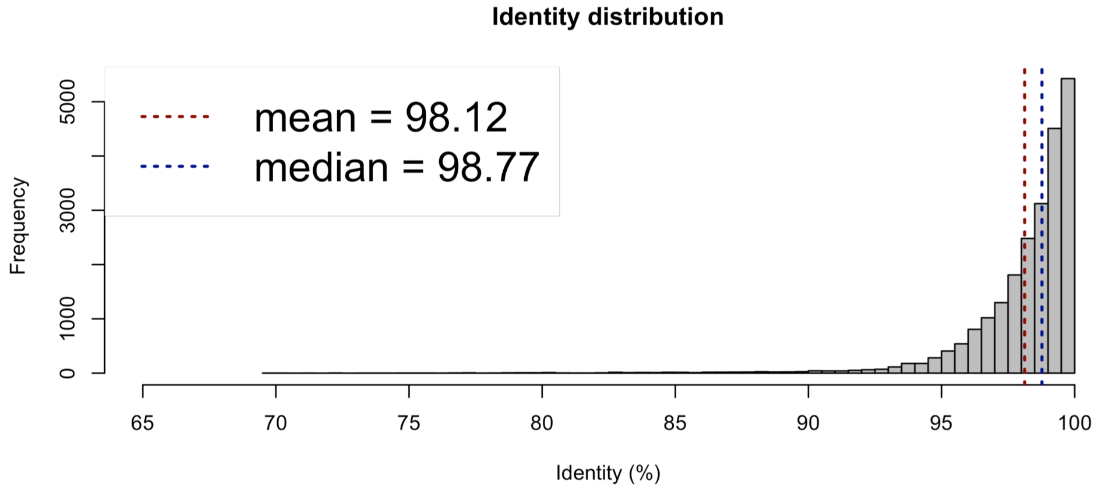

Supplementary figure 2: Estimation of two ways average nucleotide identity (ANI) between *M. llatzerense* EDP_4 and *M. llatzerense* CLUC14. Assemblies were collected, and genome to genome average nucleotide identity was determined using the online tool ANI calculator (http://enve-omics.ce.gatech.edu/ani/), with a minimum alignment length of 500 nucleotides (corresponding to the smallest contig size for *M. llatzerense* EDP_4), a window size of 500 nucleotides, and a step size of 100 nucleotides[4]. The resulting output was plotted according to the identity values on x axis, and the corresponding number of fragments (frequency). ANI calculation was based on alignment and comparison of 70% and 76% of sequences from *M. llatzerense EDP_*4 and *M. llatzerense* CLUC14 assemblies, respectively.

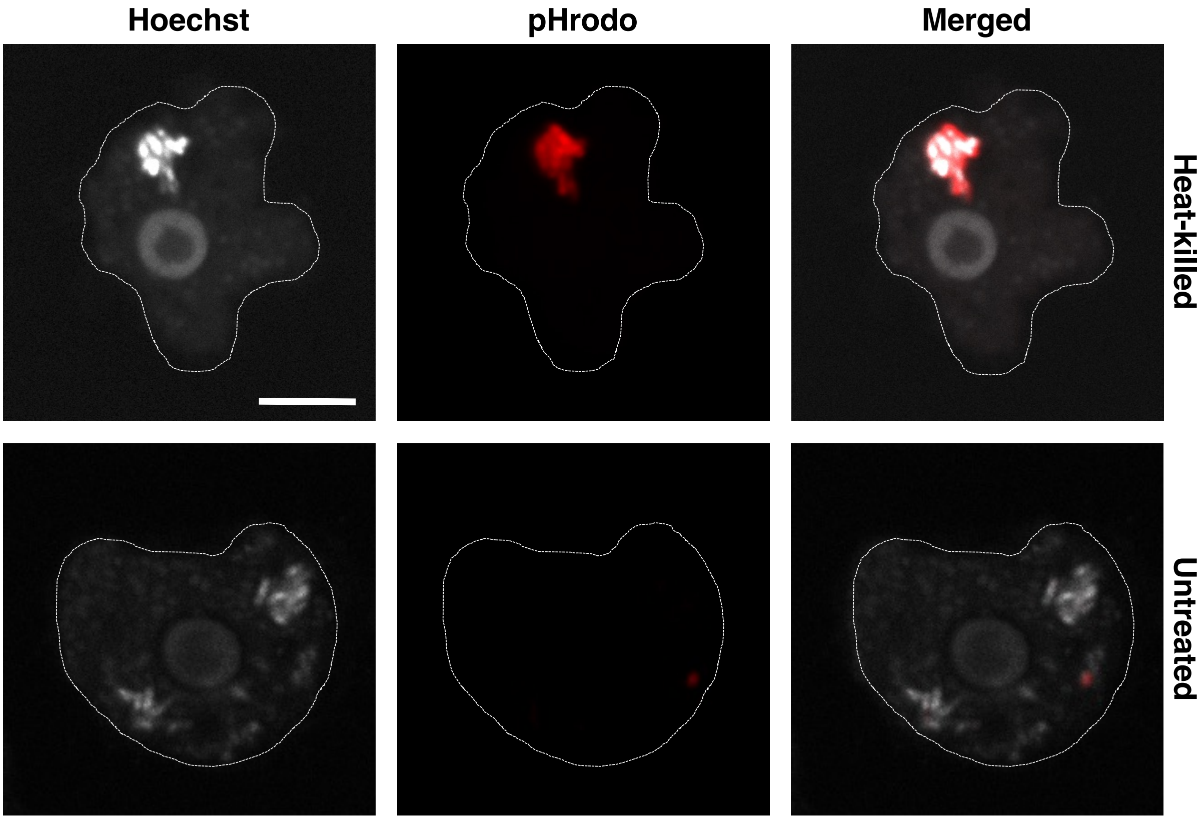

Supplementary figure 3: Assessment of *M. llatzerense* localization within intracellular acidic compartments following infection of *A.castellanii*. Amoeba cells were infected by *M. llatzerense* pre-labelled with pHrodo (red), as described in the methods, and visualised using confocal laser scanning microscope. Each micrograph was acquired using similar setting as to enable comparison of fluorescence intensity. Bar length represents 5µm.

References :

1. Carroll, P. *et al.* Sensitive detection of gene expression in mycobacteria under replicating and non-replicating conditions using optimized far-red reporters. *PLoS One* **5,** e9823 (2010).
2. Barisch, C., Paschke, P., Hagedorn, M., Maniak, M. & Soldati, T. Lipid droplet dynamics at early stages of Mycobacterium marinum infection in Dictyostelium. *Cell. Microbiol.* (2015). doi:10.1111/cmi.12437
3. Greninger, A. L. *et al.* Two Rapidly Growing Mycobacterial Species Isolated from a Brain Abscess: First Whole-Genome Sequences of Mycobacterium immunogenum and Mycobacterium llatzerense. *J. Clin. Microbiol.* **53,** 2374–7 (2015).
4. Klappenbach, J. A. *et al.* DNA–DNA hybridization values and their relationship to whole-genome sequence similarities. *Int. J. Syst. Evol. Microbiol.* **57,** 81–91 (2007).
